# Supplementary material for: Risk factors mediating the effect of body mass index and waist-to-hip ratio on cardiovascular outcomes: Mendelian randomization analysis
Source: Int J Obes (Lond). 2021 May 17;45(7):1428–38. doi: 10.1038/s41366-021-00807-4 (PMC8236409; doi:10.1038/s41366-021-00807-4)

**Supplement**

**Risk factors mediating the effect of body-mass index and waist-to-hip ratio on cardiovascular outcomes: Mendelian randomization analysis**

**Supplementary Figure 1. Total effects of genetically predicted body mass index (BMI) and genetically predicted waist-to-hip ratio (WHR) on coronary artery disease (CAD), peripheral artery disease (PAD) and stroke**. Inverse-variance weighted (IVW), contamination-mixture method (Con-Mix), weighted median (Median) and Egger represent distinct Mendelian randomization approaches that differ in their requisite assumptions. CI: confidence interval; IVW: inverse-variance weighted; OR: odds ratio; SD: standard deviation.


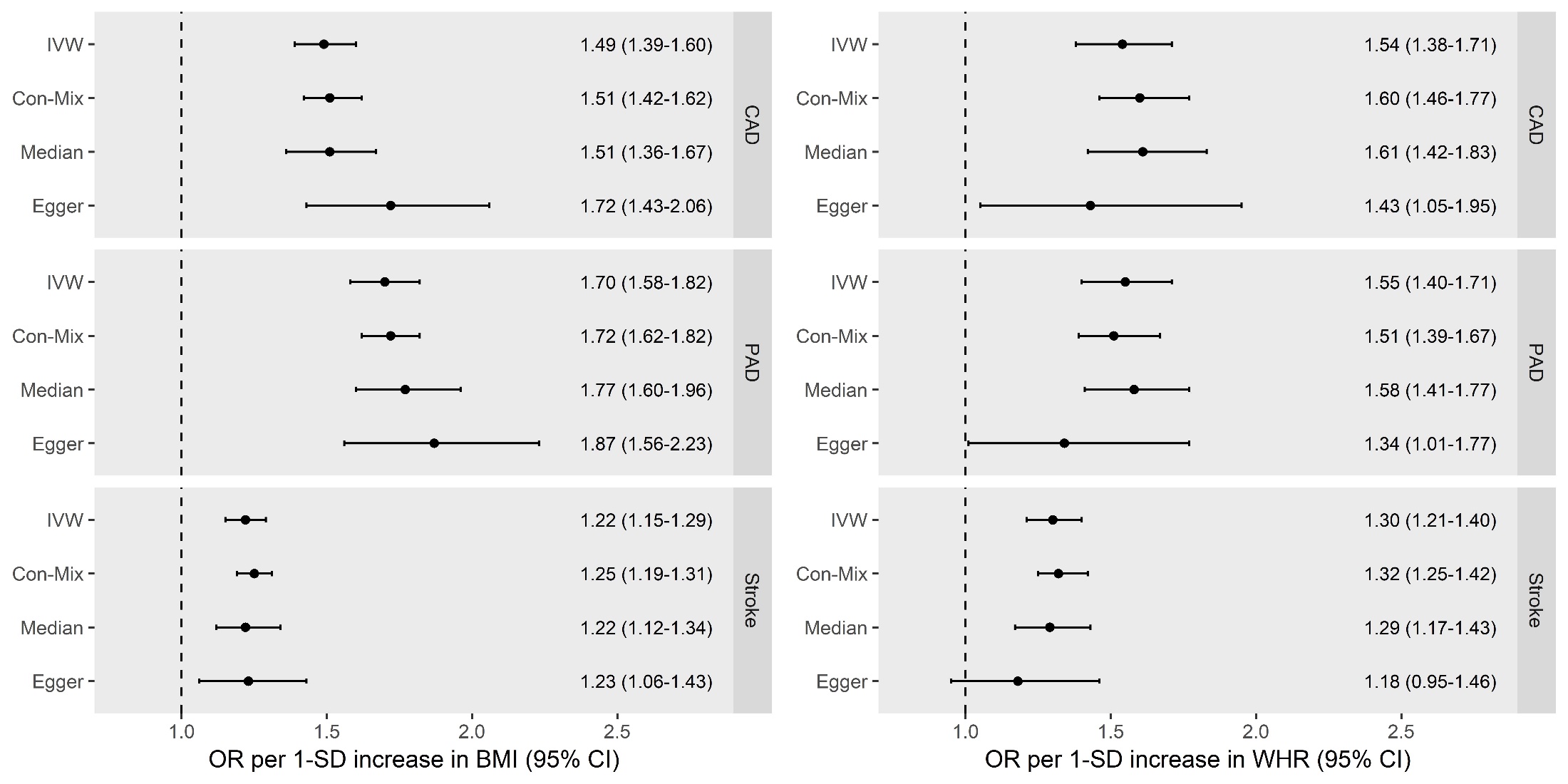


**Supplementary Figure 2. Direct effects of genetically predicted body mass index (BMI) and genetically predicted waist-to-hip ratio (WHR) on coronary artery disease (CAD), peripheral artery disease (PAD) and stroke, estimated after no adjustment and after adjusting for all considered genetically predicted mediators except diabetes (i.e. systolic blood pressure, low-density lipoprotein cholesterol, high-density lipoprotein cholesterol, triglycerides and smoking)**. CI: confidence interval; OR: odds ratio; SD: standard deviation.


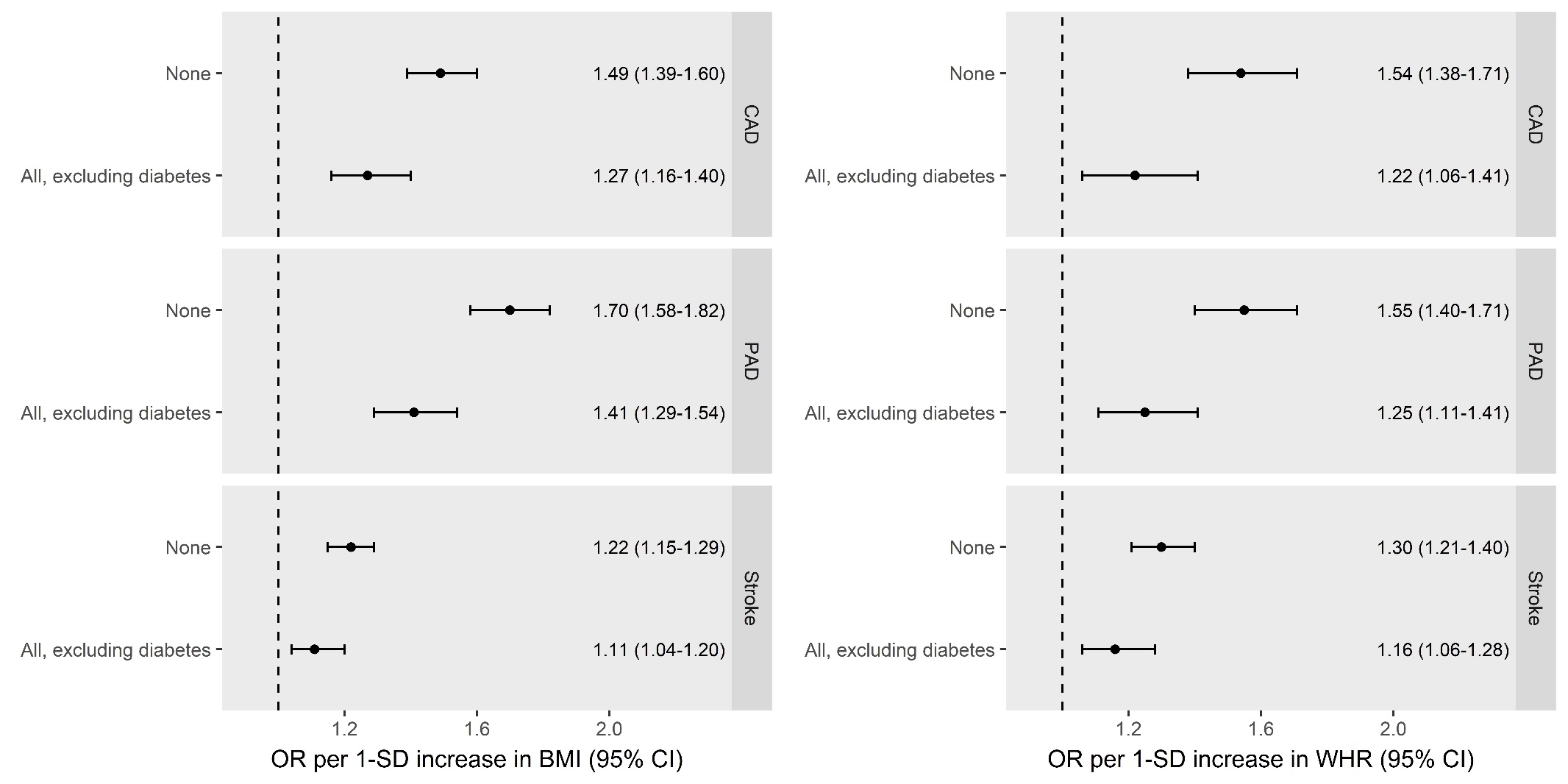

Supplement: Supplementary file 1 — Supplemental Material [file 41366_2021_807_MOESM1_ESM.docx]
